# Pathway-Based Genome-wide Association Studies Reveal That the Rac1 Pathway Is Associated with Plasma Adiponectin Levels

**DOI:** 10.1038/srep13422

**Published:** 2015-08-24

**Authors:** Wei-Dong Li, Hongxiao Jiao, Kai Wang, Fuhua Yang, Struan F. A. Grant, Hakon Hakonarson, Rexford Ahima, R. Arlen Price

**Affiliations:** 1 Research Center of Basic Medical Sciences, Tianjin Medical University, Tianjin, 300070, China; 2 Center for Neurobiology and Behavior, Department of Psychiatry, University of Pennsylvania Perelman School of Medicine, Philadelphia, PA 19104, USA; 3 Zilkha Neurogenetic Institute and Norris Comprehensive Cancer Center, University of Southern California, Los Angeles, CA 90089, USA; 4 Center for Applied Genomics, Children’s Hospital of Philadelphia, Philadelphia, PA 19104, USA; 5 Department of Pediatrics, University of Pennsylvania Perelman School of Medicine, Philadelphia, PA 19104, USA; 6 Department of Medicine, Division of Endocrinology, Diabetes and Metabolism, University of Pennsylvania Perelman School of Medicine, Philadelphia, PA 19104, USA

## Abstract

Pathway-based analysis as an alternative and effective approach to identify disease-related genes or loci has been verified. To decipher the genetic background of plasma adiponectin levels, we performed genome wide pathway-based association studies in extremely obese individuals and normal-weight controls. The modified Gene Set Enrichment Algorithm (GSEA) was used to perform the pathway-based analyses (the GenGen Program) in 746 European American females, which were collected from our previous GWAS in extremely obese (BMI > 35 kg/m^2^) and never-overweight (BMI<25 kg/m^2^) controls. Rac1 cell motility signaling pathway was associated with plasma adiponectin after false-discovery rate (FDR) correction (empirical *P* < 0.001, FDR = 0.008, family-wise error rate = 0.008). Other several Rac1-centered pathways, such as cdc42racPathway (empirical *P* < 0.001), hsa00603 (empirical *P* = 0.003) were among the top associations. The RAC1 pathway association was replicated by the ICSNPathway method, yielded a FDR = 0.002. Quantitative pathway analyses yielded similar results (empirical *P* = 0.001) for the Rac1 pathway, although it failed to pass the multiple test correction (FDR = 0.11). We further replicated our pathway associations in the ADIPOGen Consortium data by the GSA-SNP method. Our results suggest that Rac1 and related cell motility pathways might be associated with plasma adiponectin levels and biological functions of adiponectin.

Adiponectin is an adipocyte-specific secretory protein, which is associated with type 2 diabetes mellitus (T2DM), obesity, dyslipidemia, and other insulin-resistance related phenotypes. With the development of genome-wide association studies (GWAS), more than a dozen genes were found to be associated with adiponectin. The R112C mutation in the third exon of the adiponectin gene was significantly associated with lower plasma adiponectin levels^1^. Some of those genes were replicated in different populations, including *ADIPOQ*, *ARL15*, and *CDH13*^2^,^3^,^4^,^5^. Adiponectin levels were correlated with body mass index (BMI), yet only a few genes were associated with both BMI and adiponectin. Although, many studies suggested that adiponectin connects obesity, glucose metabolism, and inflammation, the genetic background of that connection remains enigmatic.

During the past two decades, we have been working on genetics of human obesity in families segregating extremely obese and normal-weight siblings^6^,^7^,^8^,^9^. After plasma adiponectin levels were measured in our subjects, we performed a GWAS for plasma adiponectin in extremely obese cases and in unrelated normal-weight controls.

Although the intensive search for adiponectin-related genes by GWAS was successful in many studies, we still could not explain the heritability of adiponectin by several top candidate genes. On the other hand, an enormous number of genes were only marginally associated with adiponectin. Many those genes might affect major pathways that play important roles in biological functions of adiponectin. To identify the genes and signaling pathways associated with plasma adiponectin, we further performed pathway-based association analyses in extremely obese individuals and normal-weight controls based on our adiponectin GWAS data.

## Results

### Genome-wide Association Studies

We firstly performed a quantitative GWAS for adiponectin in 737 females. We found that the *ADIPOQ* gene was associated with BMI-adjusted adiponectin (rs822387, *P* = 3.77 × 10^−7^). Weaker associations were found for *DLEU1* (rs200220, *P* = 7.49 × 10^−7^), *ALK* (rs13029602, *P* = 7.48 × 10^−6^), and *CREG2* (rs4352251, *P* = 1.86 × 10^−6^). Associations with *P* < 1 × 10^−5^ are shown in Table 1. No genome-wide significance was reached (P < 1 × 10^−7^, given the 550K SNPs in the genotyping panel).

### Pathway Association Studies

Pathway-based association studies (GenGen) were carried out for plasma adiponectin, in the case/control analysis, 74 pathways achieved a significance of empirical *P* < 0.05; however, only the Rac1 pathway (empirical *P* < 0.001, FDR = 0.008, FWER = 0.008) passed correction for multiple testing. *RAC1* was present in both Rac1 and Cdc42/Rac pathways (empirical *P* < 0.001, FDR = 0.188, FWER = 0.249; Table 2).

In the quantitative pathway-based association study, 61 pathways associated with BMI-adjusted adiponectin and 71 were associated with log-adiponectin (empirical *P* < 0.05). These pathways contained many genes related to T2DM and insulin resistance. Genes of the RAS and downstream PI3K/AKT and MAPK/ERK signaling pathways were found in multiple significant pathways, including TID, RNA, hsa04150, actin y, Par1, and GH pathways. The RAS signaling pathway was associated with adiponectin after FDR correction (empirical *P* < 0.001, FDR = 0.01; Table 2). *RAC1* was also present in the hsa05030 (empirical *P* = 0.048), MAL (empirical *P* = 0.040), and actin y (empirical *P* = 0.019) pathways. Multiple complement-activation-related pathways were also associated with adiponectin levels (empirical P = 0.001).

Seventy-five (75) pathways yielded significant empirical *P*-values (empirical *P* < 0.05) in the quantitative pathway-based association study for unadjusted adiponectin. The *RAC1* gene was present in the GO0032270 (empirical *P* = 0.011) and GO0051247 (empirical *P* = 0.011) pathways, although none of these pathways remained significant after correction for multiple testing.

Seventy pathways were associated with BMI in extremely obese cases and nominal-weight controls (empirical *P* < 0.05); however, none of them reached FDR < 0.05 after correcting for multiple tests.

The RAC1 pathway association was replicated by the ICSNPathway assay. The RAC1 pathway yielded a FDR = 0.002 (empirical P < 0.001) for binary adiponectin.

Pathway association studies were also performed for body weight related phenotypes, including BMI, %fat, waist and hip circumferences, waist/hip ratio, and A Body Shape Index (ABSI). None of these analyses reached FDR < 0.05 by GenGen. Besides, the Rac1 pathway was not among top pathways (ranked by empirical *P* values) that associated with body weight related phenotypes.

We further replicated our results in the ADIPOGen Consortium. Using SNP specific *P* values from the ADIPOGen GWAS, we performed pathway association studies by GSA-SNP. A key pathway found in our study, GO0032990 (centered by the *Rac-1* pathway and the *CDH13* gene) was among the top associations (corrected *P*  = 1.06 × 10^−13^, FDR = 0) in the ADIPOGen data (Supplement Information).

Since no phenotype (adiponectin) was available online from the ADIPOGen Consortium data^10^, we could not test pathway associations for the ADIPOGen data by GenGen. To further verify our results, we have conducted GSA-SNP^11^ pathway association tests using our GWAS data. *Rac-1* and related pathways were still among the most significant associations, while pathways that harbored *CDH13* showed significant results as in the ADIPOGen data (Supplement Information). Forty-nine (49) pathways yielded significant associations (corrected *P* < 0.05, FDR = 0), 12 of 49 pathways were *Rac-1* related.

## Discussion

In this pathway-based GWASs, we found downstream branches of the RAS signaling pathway, including the PI3K/AKT, MAPK/ERK, and Rac1 cell motility signaling pathways, were associated with plasma adiponectin levels. Among them, the Rac1 cell motility signaling pathway was associated with adiponectin in both case/control and quantitative genome-wide pathway association studies. We found many T2DM susceptibility genes in adiponectin-related pathways, but only a few of them were present in BMI-related pathways. These data suggest that adiponectin may play a role in connecting obesity and T2DM.

So far, more than a dozen GWASs on adiponectin have been carried out, and at least 15 loci have been associated with plasma adiponectin levels^12^. *ADIPOQ*, *ARL15*, and *CDH13* are among the most replicated associations^2^,^3^,^4^. Moreover, polymorphisms on *FER*^13^, *ETV5*^14^, and *KNG1*^15^ have been associated with plasma adiponectin. Dastani *et al.*^10^ performed meta-analysis of adiponectin GWAS in 45891 individuals (The ADIPOGen Consortium), *ADIPOQ* yielded the most significant association. *ADIPOQ* gene had among the strongest associations in our GWAS. SNPs rs822387 and rs17300539 reached significance (*P* < 10^−5^) for both quantitative adiponectin and BMI-adjusted adiponectin. We also found *ADIPOQ* in the obesity (empirical *P* = 0.019) and hsa04930 (empirical *P* = 0.027) pathways associated with BMI-adjusted adiponectin. The interactions among *ADIPOQ* and other genes in these pathways may contribute to the association with adiponectin. Hivert^16^ and Schwarz *et al.*^17^ found that *ADIPOQ* polymorphisms were associated with plasma adiponectin levels and T2DM. The promoter region of *ADIPOQ* has C/EBP and PPARγ binding sites, and the biological interactions between *ADIPOQ* and C/EBP are well documented^18^.

*CDH13* is another adiponectin-related gene, although *CDH13* was not significantly associated with adiponectin in our GWAS, it was present in several pathways that associated with adiponectin, including GO0045296, GO0030100, and GO0007156.

Although “top” associations may explain some of the genetic variation in plasma adiponectin levels, many minor associations contributed the majority of genetic background. Given the relatively limited sample size and genetic relative risk, those gene polymorphisms are unlikely to be identified by GWAS. However, many of those genes are clustered in certain pathways that are jointly associated with adiponectin.

In our study, we used modified GSEA (GenGen) to identify pathways for both binary (case/control) and quantitative adiponectin, and found the Rac1 pathway was the most significant related pathway. In this pathway, *RAC1* plays an important role in cell migration via its regulation of actin filament organization^19^. In the Cdc42/Rac pathway (*P* < 0.001), N-WASP activates ARP-2/3 for actin polymerization and filopodium formation^20^. The function of N-WASP is dependent on the formation of the RAC-IRSP53-WAVE2 trimolecular complex. Nakamura *et al.*^21^ found that adiponectin increased the phosphorylation of Akt and activated Cdc42 and Rac1 in endothelial progenitor cells (EPCs). Interestingly, in our study the PI3K-Rac1 pathway was associated with plasma adiponectin levels and remained significant after FDR correlation. We need to point out that the “nominal” *P* values obtained by the GenGen analysis were actually empirical *P* values: it was calculated by comparing the pathway enrichment score versus 1000 times phenotypes permutation, it was far more conservative than nominal *P* values in association studies. It was rare to have significant FDR in both GenGen and ICSNPathway.

*RAC1* harbored in many pathways that associated with adiponectin and BMI-adjusted adiponectin, which suggests that *RAC1* could be a key connector between adiponectin and its biological functions in cell adhesion, migration, and inflammation. Syed *et al.*^22^ provided evidence of a direct connection among hyperglycemia, Rac1 activation, and β-cell apoptosis: Rac1 expression was significantly increased in human islets after exposure to 30 mmol/L glucose; Rac1-Nox-ROS signaling led to caspase 3 activity and mitochondrial dysregulation. It worth further studies on whether Rac1 also mediated adiponectin related cell survival and apoptosis.

In our quantitative pathway association study, many downstream pathways of the RAS signal transduction, including PI3K/AKT, MAPK/ER, NF-κB (nuclear factor κB), and mTOR (mammalian target of rapamycin), were associated with adiponectin. The PI3K/AKT pathway is well studied in insulin signal transduction. TNF-α (tumor necrosis factor-α) and IL-6 (interleukin-6) activate the PI3K/AKT pathway to induce cell growth and maintain cell survival, whereas adiponectin inhibits TNF-α and IL-6 to regulate the PI3K/AKT pathway^23^. Indeed, adiponectin inhibits TNF-α expression in macrophages^24^. Ouchi *et al.*^25^ showed that adiponectin stimulated angiogenesis by promoting cross-talk between AMP-activated protein kinase and Akt signaling.

NF-κB is a nuclear factor that plays key roles in apoptosis, immune response, and inflammation and can be activated by TNF-α and IL-1β. Adiponectin could inhibit TNF-α and thereby suppress the activity of NF-κB and expression of adhesion factors. The anti-inflammatory effect of adiponectin is related to its inhibition of NF-κB through the PI3K/AKT pathway^26^,^27^; Kobashi *et al.*^28^ indicated that adiponectin inhibits NF-κB via the PI3K/AKT pathway and thereby regulates inflammatory factors in endothelial cells.

mTOR is an important downstream serine/threonine protein kinase of the PI3K/AKT pathway. It has profound biology effects on ribosome syntheses, gene transcription, protein translation, inflammation, apoptosis, and cell proliferation and differentiation. Many studies have demonstrated that adiponectin was the key mediator between mTOR and its biological effects^29^. Sugiyama *et al.*^30^ also found that adiponectin inhibits colorectal cancer cell growth through the AMPK/mTOR pathway.

The MAPK/ERK pathway involved in cell proliferation, differentiation, and apoptosis has profound connections with inflammation and carcinogenesis. Although this study was the first to associate the MAPK/ERK pathway with adiponectin, many other studies have suggested that MAPK/ERK mediates biological effects of adiponectin: adiponectin regulates wound healing by promoting keratinocyte proliferation and migration via the ERK pathway^31^. Li *et al.*^32^ found that adiponectin upregulates prolyl-4-hydroxylase α1 expression in aortic smooth muscle cells via ERK1/2 and Sp1.

All PI3K/AKT, MAPK/ERK, and Rac1 pathways are lower branches of the RAS signal transduction pathway (http://www.biocarta.com/pathfiles/h_rasPathway.asp), it is suggested that the RAS pathway might be associated with adiponectin. We tested a cluster of RAS pathway genes (Table 3) with GenGen and found a significant association for BMI-adjusted adiponectin (empirical *P* < 0.001, FDR = 0.01). Ample research has indicated that almost all “branches” of the RAS signaling pathway mediate biological effects of adiponectin.

Many T2DM susceptibility genes, including *PPARG*, *CDKN2A/B* (*ANRIL*), and *KCNJ11*, are found in key adiponectin-related pathways identified in this study. However, only a few of these genes are present in pathways associated with BMI. The connections between adiponectin and T2DM, inflammation, and cancer are well studied; it would be interesting to examine how many T2DM and inflammation-related genes and pathways were actually associated with plasma adiponectin levels in the obese population.

Although pathway association studies may less likely to pick up false positive results than in single-SNP associations, replication in separate cohort is needed. We tested our pathway associations in a much larger data set, the ADIPOGen Consortium. Not to our surprise, *CDH13* and *ADIPOQ* centered pathways showed top associations. Interestingly, a *Rac-1* related pathway is among the most significant pathway associations. Similarly, *Rac-1* related pathways dominated the GSA-SNP association list in our data set. Actually, *Rac-1* pathways were among top associations in both binary and quantitative assays and in all 3 different tests using phenotype or SNP specific *P* values permutations (GenGen, ICSNPathway, and GSA-SNP). The most important, the *Rac-1* pathway association was replicated in different populations.

The *Rac-1* gene association was not among the top associations in GWASs, however, *Rac-1* related pathways were significantly associated with plasma adiponectin levels. Needless to say, more studies are needed to decipher the connections between these pathway associations and the biological effects of adiponectin.

## Materials and Methods

### Subjects

We collected 1,071 unrelated European Americans: 526 extremely obese (BMI > 35 kg/m^2^) and 545 normal-weight controls (BMI < 25 kg/m^2^)^9^. Subjects were originally collected and genotyped for a GWAS for obesity^9^. Of these, we had adiponectin data for 764, 746 of which were females. In this study, we performed our analyses only in females. Adiponectin outliers (>3 SD) were excluded from the data set, 9/746 samples were removed, (see Table 4).

All subjects gave informed consent, and the protocol was approved by the Committee on Studies Involving Human Beings at the University of Pennsylvania. The study was carried out in accordance with the approved guidelines. Plasma adiponectin levels were detected by radioimmunoassay by the Obesity Unit of the Institute for Diabetes, Obesity and Metabolism at the Perelman School of Medicine, University of Pennsylvania.

### Genome-wide Association Study

**Figure 1:**
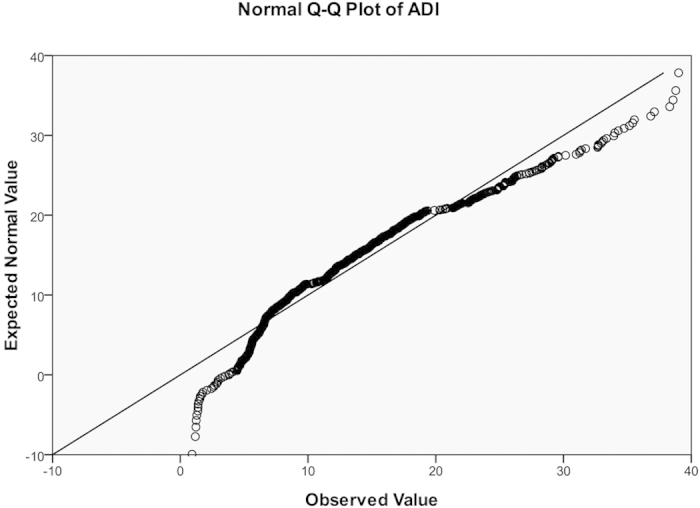
Q-Q plots for distributions of adiponectin.

**Figure 2:**
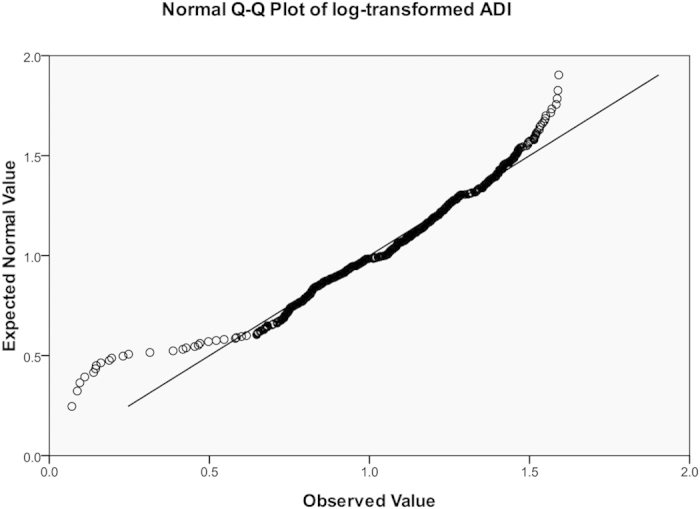
Q-Q plots for distributions of log-transformed adiponectin.

**Figure 3:**
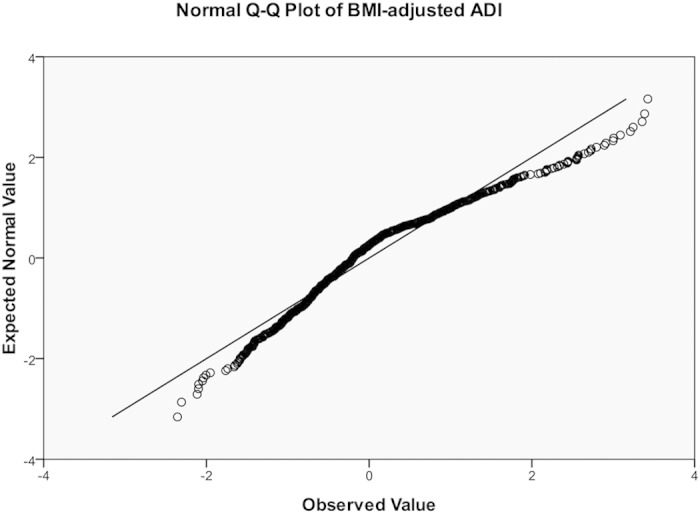
Q-Q plots for distributions of BMI-adjusted adiponectin.

We genotyped 550,000 single-nucleotide polymorphisms (SNPs) by Illumina HumanHap 550 SNP Arrays in our previous GWAS for obesity^9^. Genome-wide association analyses for adiponectin were carried out by PLINK (http://pngu.mgh.harvard.edu/~purcell/plink/)^33^, the values of Minor Allele Frequency (MAF) and Hardy-Weinberg equilibrium(HWE) were defaulted. Since subjects were originally recruited for an obese case-control study, plasma adiponectin levels were adjusted by BMI: linear regressions were performed for adiponectin against BMI and standardized residuals were saved as “BMI-adjusted adiponectin” for association studies (mean = 0, standard deviation = 1). Both original, log transformed, and BMI-adjusted adiponectin data were used for quantitative association studies. Outliers (>±3 SD) were deleted before analysis. Distributions of adiponectin, log-transformed, and BMI-adjusted adiponectin were shown as Figs 1, 2, 3 and Table 4.

### Pathway Association Study

Pathway-based association studies were performed using the GenGen program^34^, with calculations based on the modified Gene Set Enrichment Algorithm (GSEA)^35^. Briefly, For each gene, the SNP with the highest test statistic (chi-square detection/*F*-test) among all SNPs mapped to the gene was selected to represent the gene. All genes were ranked by sorting their statistic values from the largest to smallest, denoted by *r*(1), …, *r*(*N*). For any given gene set *S* composed of *N*_*H*_ genes, an enrichment score (ES) was calculated,


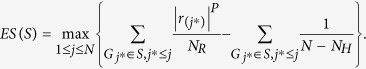


Where 
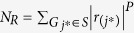
 and *p* is a parameter that gives higher weight to genes with extremes statistic values (default *p* = 1). Then, in order to adjust the size of different genes, phenotype label permutation with 1000 permutations was performed, and for each permutation, all the above calculations were repeated. We then calculated the normalized enrichment score (*NES*): 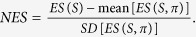


Finally, a false-discovery rate (FDR) procedure was conducted to control the fraction of expected false-positive findings: 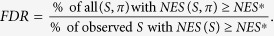


Where NES* denotes the normalized ES in the observed data.

A total of 1,347 pathways compiled from the BioCarta, Kyoto Encyclopedia of Genes and Genomes (KEGG), and Gene Ontology (GO) databases were analyzed. SNPs with minor allele frequencies <0.01 and Hardy-Weinberg equilibrium <0.001 were deleted from the study. Thus, we used 518,230 SNPs in 17,437 genes for GenGen analyses (http://www.openbioinformatics.org/gengen/), 20 k base pairs upstream and downstream of each gene was considered to be a part of the gene.

In dichotomous “case-control” pathway association studies, we defined individuals with plasma adiponectin >10.4 mg/L as “cases” and those with plasma adiponectin <7.4 mg/L as “controls” (Table 5). The “cases” and “controls” were defined by a dichotomous threshold (with a gray area in between) rather than an affection status. In quantitative analyses, we deleted outliers with >±3 SD. Plasma adiponectin levels were also adjusted by BMI: linear regression was performed for adiponectin against BMI, and standardized residuals were saved such that mean = 0 and SD = 1. Quantitative pathway association studies (GenGen) were performed for original, log transformed, and BMI-adjusted adiponectin.

Since pathway association studies showed multiple RAS-pathway-related associations (see **Results**), we further tested a cluster of genes in the RAS pathway by GenGen for both quantitative and discrete adiponectin. We also conducted pathway association studies for binary BMI in the extremely obese cases and normal-weight controls.

### Pathway association studies by ICSNPathway

After we got the results from GenGen, we further tested the pathway associations by ICSNPathway (http://icsnpathway.psych.ac.cn/)^36^. ICSNPathway took *P* values of each SNP that obtained from GWAS, however, no phenotype permutation was performed. ICSNPathway analyses were performed for case/control data, SNPs with *P* < 0.01 (PLINK for binary adiponectin GWAS) and r^2^ > 0.6 were selected in our study.

### Replication of the pathway association results in ADIPOGen Consortium

We further replicated our pathway associations using the ADIPOGen Consortium data^10^ (http://www.mcgill.ca/genepi/adipogen-consortium). We obtained SNP specific *P* values from the ADIPOGen GWAS for 45891 individuals, GSA-SNP^11^ tests were performed to identify pathways that associated with plasma adiponectin levels. GSA-SNP permutated SNP specific *P* values other than the phenotype (adiponectin) used in GenGen. To better compare ADIPOGen pathway associations with ours, we also conducted GSA-SNP pathway association studies in our binary adiponectin data that used in GenGen.

## Conclusions

In this study, we found significant associations between the Rac1 pathway and the plasma adiponectin level in extremely obese individuals and normal-weight controls. Genome-wide pathway association studies (GenGen) yielded similar results in binary and quantitative analyses. The Rac-1 association was further verified using the ICSNPathway method. It is suggest that Rac1 and related cell motility pathways might be associated with plasma adiponectin levels and biological functions of adiponectin.

## Additional Information

**How to cite this article**: Li, W.-D. *et al.* Pathway-Based Genome-wide Association Studies Reveal That the Rac1 Pathway Is Associated with Plasma Adiponectin Levels. *Sci. Rep.* **5**, 13422; doi: 10.1038/srep13422 (2015).

## Supplementary Material

Supplementary Information

## Figures and Tables

**Table 1:** Genome-wide association studies for quantitative plasma adiponectin.

**Chr**	**SNP**	**BP**	**MAF**	**Gene**	***P*** **(ADI)**	***P*** **(BMI-ADI)**	***P*****(Log-ADI)**	ADIPOGen Consortium^10^(*P*values)
13	rs200220	49858379	0.122	DLEU1	7.29 × 10^−6^	7.49 × 10^−7^	1.74 × 10^−5^	0.0120
13	rs188014	49867924	0.116	DLEU1	5.36 × 10^−6^	7.85 × 10^−7^	1.65 × 10^−5^	0.2278
3	rs822387	188038731	0.077	ADIPOQ	8.39 × 10^−6^	3.77 × 10^−7^	1.03 × 10^−4^	0
3	rs17300539	188042154	0.076	ADIPOQ	1.46 × 10^−4^	6.14 × 10^−6^	9.61 × 10^−4^	0
2	rs13029602	29428379	0.371	ALK	4.95 × 10^−5^	7.48 × 10^−6^	5.32 × 10^−5^	0.3367
2	rs4352251	101350094	0.111	CREG2	2.51 × 10^−6^	1.86 × 10^−6^	4.26 × 10^−5^	0.8026
2	rs10497399	173822385	0.075	ZAK	2.37 × 10^−6^	4.00 × 10^−4^	3.15 × 10^−5^	0.8006
2	rs6731322	173825266	0.103	ZAK	2.28 × 10^−5^	1.24 × 10^−2^	1.07 × 10^−4^	0.6263
10	rs2447014	14050128	0.372	FRMD4A	2.26 × 10^−5^	1.32 × 10^−3^	1.72 × 10^−4^	0.4372
10	rs4750440	14053960	0.397	FRMD4A	5.60 × 10^−6^	7.57 × 10^−4^	6.17 × 10^−5^	0.5617
10	rs10508470	14061828	0.308	FRMD4A	8.82 × 10^−5^	2.98 × 10^−3^	4.63 × 10^−4^	0.9373
12	rs11832828	42881817	0.088	TMEM117	4.46 × 10^−5^	1.66 × 10^−4^	1.88 × 10^−4^	0.4925
12	rs7961974	42890295	0.096	TMEM117	2.26 × 10^−5^	4.90 × 10^−5^	8.36 × 10^−5^	0.5429
13	rs7327245	23698063	0.336	SPATA13	3.24 × 10^−5^	5.66 × 10^−5^	9.70 × 10^−5^	0.5228
13	rs9578695	23699309	0.325	SPATA13	4.17 × 10^−5^	8.00 × 10^−5^	1.94 × 10^−4^	0.3563
17	rs2377301	74505964	0.100	CANT1	8.41 × 10^−5^	5.26 × 10^−4^	8.02 × 10^−4^	0.5259
17	rs4789848	74514952	0.132	CANT1	9.66 × 10^−5^	2.18 × 10^−4^	5.52 × 10^−4^	0.9170
18	rs3813086	46442790	0.253	MAPK4	9.32 × 10^−5^	9.40 × 10^−5^	3.24 × 10^−4^	0.4497
18	rs3911593	46436326	0.265	MAPK4	1.67 × 10^−4^	8.88 × 10^−5^	4.32 × 10^−4^	0.5883
18	rs4260159	46483873	0.130	MAPK4	7.03 × 10^−5^	3.06 × 10^−3^	2.25 × 10^−4^	0.9807

MAF, minor allele frequency; ADI, adiponectin; BMI-ADI, BMI-adjusted adiponectin; Log-ADI, log-transformed adiponectin.

**Table 2:** Pathway association study for adiponectin and BMI-adjusted adiponectin.

**Pathway ID**	**genes**	**empirical** ***P***	**FDR**	**FWER**	**database**
Adiponectin (binary)					
Rac1	23	<0.001	0.008	0.008	BioCarta
Cdc42/Rac	15	<0.001	0.188	0.249	BioCarta
GO0042287	13	<0.001	0.884	1.000	GO
Log-adiponectin Rac1	23	0.009	0.685	0.409	BioCarta
BMI-adjusted adiponectin (quantitative)					
RAS	43	<0.001	0.01	0.01	Self-defined*

^*^A cluster of RAS pathway genes were selected based on the pathway association study (listed in Table 3).

**Table 3:** RAS pathway genes tested for associations with BMI-adjusted plasma adiponectin.

**RAS pathway genes (empirical** ***P***** < 0.001, FDR = 0.01)**								
ACTA1	BCL2	CASP8	FRAP1	CDK5RAP2	PIK3CA	PIK3R3	MAPK3	VAV1
AKT1	BCL2L1	CASP9	FRAP2	MAP2K1	PIK3CB	PIK3R5	MAPK8	VAV2
AKT2	BRAF	CDC42	HRAS	MAP2K2	PIK3CD	RALBP1	NFKB1	VAV3
AKT3	CASP1	SOS2	LIMK1	MAP4K2	PIK3CG	WASF1	PTPN6	
ARFIP2	CASP10	CFL1	CDK5R1	NFKBIA	PIK3R1	RHOA	RAC1	

**Table 4:** Distribution of plasma adiponectin in all subjects (all females).

	**N**	**max**	**min**	**mean**	SD*	**skewness**	**kurtosis**
Adiponectin (mg/L)	737	39.01	1.18	13.97	7.60	0.85	0.37
Log-adiponectin	737	1.59	0.07	1.07	0.26	−0.74	1.16
BMI-adiponectin**	737	2.97	−2.12	0	1.00	0.86	0.79

^*^Outliers (>±3 SD) were deleted in this study.

^**^Adiponectin adjusted by BMI.

**Table 5:** Distribution of plasma adiponectin in “cases” and “controls”.

	**N**	**age**	**adiponectin** (**mg/L**)	**BMI** > **35** **kg/m**^**2**^	**BMI** < **25** **kg/m**^**2**^	**max**	**min**	**mean**	**SD**
Cases	474	43.74 ± 8.49	>10.4	88	386	51.98	10.42	18.74	7.37
Controls	165	40.68 ± 8.81	<7.4	130	35	7.30	1.18	5.47	1.59
