# Supplementary material for: Pathway-Based Genome-wide Association Studies Reveal That the Rac1 Pathway Is Associated with Plasma Adiponectin Levels
Source: Sci Rep. 2015 Aug 24;5:13422. doi: 10.1038/srep13422 (PMC4642532; doi:10.1038/srep13422)
Supplement: Supplementary Information [file srep13422-s1.pdf]

Pathway-Based Genome-wide Association Studies Reveal That the Rac1 Pathway Is Associated with Plasma Adiponectin Levels

Running title: Rac1 pathway is associated with adiponectin levels

Wei-Dong Li[1,2*#], Hongxiao Jiao[1#], Kai Wang[3#], Fuhua Yang[1], Struan F.A. Grant[4,5], Hakon Hakonarson[4,5], Rexford Ahima[6], and R. Arlen Price[2*]

1. Research Center of Basic Medical Sciences, Tianjin Medical University, Tianjin, 300070, China 2: Center for Neurobiology and Behavior, Department of Psychiatry, University of Pennsylvania Perelman School of Medicine, Philadelphia, PA 19104, USA 3. Zilkha Neurogenetic Institute and Norris Comprehensive Cancer Center, University of Southern California, Los Angeles, CA 90089, USA 4: Center for Applied Genomics, Children's Hospital of Philadelphia, Philadelphia, PA 19104, USA 5: Department of Pediatrics, University of Pennsylvania Perelman School of Medicine, Philadelphia, PA 19104, USA 6. Department of Medicine, Division of Endocrinology, Diabetes and Metabolism, University of Pennsylvania Perelman School of Medicine, Philadelphia, PA 19104, USA

#These authors contributed equally to this work

**: Correspondence should be addressed to:*

Wei-Dong Li, M.D., Ph.D.

Email: liweidong98@tijmu.edu.cn

Tel: 86-22-8333-6586

Fax: 86-22-8333-6586

and

R. Arlen Price, Ph.D.

Email: arlen@exchange.upenn.edu

Tel: 215-898-0214

Fax: 215-573-2041

Supplement Table 1 GSA-SNP pathway association tests for binary adiponectin (corrected *P*<0.05, FDR=0)

pathways*	gene count	set size	*P*	corrected *P*	FDR	gene symbols**				
GO0005088	73	256	1.79E-11	1.58E-08	0	ITSN1	RAPGEF5	MCF2L2	DNMBP	PLEKHG1
hsa04510	185	288	8.91E-09	1.73E-06	0	MAPK10	TNR	COL4A4	**RAC1**	MYLK
GO0002474	17	131	4.41E-09	1.95E-06	0	B2M	TAP2	HFE	HLA-H	HLA-A
GO0051056	186	747	4.52E-09	1.95E-06	0	ITSN1	RAPGEF5	MCF2L2	RASA2	**RAC1**
hsa04730	72	132	5.74E-07	5.57E-05	0	ITPR2	PRKG2	PPP2R2C	ITPR1	GRM5
hsa04512	86	128	7.64E-07	5.57E-05	0	TNR	GP1BB	COL4A4	HSPG2	ITGB3
hsa04020	161	240	3.06E-06	1.48E-04	0	GNA14	ITPR2	PDE1C	GNAL	ITPR1
hsa04514	121	215	3.69E-06	1.48E-04	0	HLA-DOB	PTPRM	CD4	CNTNAP2	NCAM1
hsa02010	40	74	2.75E-05	8.89E-04	0	TAP2	ABCC4	ABCC6	ABCA1	ABCA4
hsa04720	65	102	4.85E-05	0.0013	0	RPS6KA2	ITPR2	ITPR1	GRM5	BRAF
GO0010324	16	48	9.06E-06	0.0020	0	GULP1	ELMO1	MYO7A	ABCA1	PLD2
hsa04530	129	187	9.61E-05	0.0023	0	ZAK	EXOC4	PARD6G	PARD3	PRKCE
hsa04520	76	111	2.04E-04	0.0044	0	PTPRM	PARD3	**RAC1**	CTNNA2	VCL
nkcellsPathway	18	21	1.99E-05	0.0054	0	B2M	**RAC1**	SYK	PIK3R1	HLA-A
hsa04360	120	207	3.61E-04	0.0070	0	SLIT3	LRRC4C	SEMA6D	**RAC1**	CDC42
hsa04664	74	123	3.63E-04	0.0070	0	MAPK10	PRKCE	**RAC1**	SYK	PRKCA
hsa04650	115	185	4.86E-04	0.0079	0	**RAC1**	IFNGR2	BRAF	SYK	PRKCA
GO0019226	97	250	4.70E-05	0.0083	0	KCNMA1	CNTNAP2	COL4A4	KCNMB4	DRD1
hsa04912	94	164	5.86E-04	0.0087	0	MAPK10	ITPR2	ITPR1	PLD2	CDC42
hsa04940	37	71	6.06E-04	0.0087	0	HLA-DOB	HLA-DRB1	PTPRN2	HLA-A	HLA-F
GO0051319	5	13	5.98E-05	0.0088	0	FOXN3	BLM	KPNA2	CENPF	GTSE1
agrPathway	33	37	6.79E-05	0.0092	0	NRG3	NRG1	**RAC1**	CHRM1	LAMA3
**GO0032990**	163	414	8.78E-05	0.0111	0	PARD3	**CDH13**	**RAC1**	PCM1	BAI1

GO0008484	12	53	8.86E-05	0.0111	0	SULF2	ARSA	SULF1	GNS	ARSB
hsa04012	82	135	9.04E-04	0.0117	0	MAPK10	NRG3	NRG1	BRAF	PRKCA
GO0050806	6	15	1.19E-04	0.0117	0	COL4A4	DRD1	GRIK2	LAMA2	CHRNB2
hsa00770	16	22	0.0010	0.0122	0	DPYD	ENPP1	VNN1	BCAT1	PANK3
hsa04070	75	107	0.0012	0.0134	0	ITPR2	ITPR1	DGKG	DGKI	PRKCA
hsa04670	104	171	0.0012	0.0134	0	**RAC1**	CTNNA2	VCL	RASSF5	MYLPF
GO0007156	82	343	1.95E-04	0.0172	0	CDH20	PCDH20	CLSTN2	**CDH13**	FAT3
GO0005513	7	17	2.25E-04	0.0181	0	KCNMB4	SYT1	KCNMB2	KCNIP1	KCNMB3
GO0030295	12	32	2.38E-04	0.0181	0	NRG3	MAPK8IP2	NRG1	TOM1L1	ERBB3
GO0007010	135	402	2.87E-04	0.0196	0	SVIL	SPTBN5	PYY	**RAC1**	DOCK2
GO0030201	12	26	3.31E-04	0.0209	0	SULF2	NDST2	CSGALNACT1	SULF1	HS3ST4
plateletAppPathway	11	15	3.21E-04	0.0289	0	COL4A4	APP	COL4A1	COL4A3	PLAU
erbB4pathway	7	8	3.34E-04	0.0289	0	NRG3	PRKCA	PRKCB	ERBB4	NRG2
keratinocytePathway	46	47	4.94E-04	0.0289	0	PRKCE	ETS1	PRKCA	RAF1	MAPK14
**GO0042287**	12	45	5.86E-04	0.0346	0	TAP2	CD4	(LAG3)	DERL1	TAPBP
GO0030165	21	44	5.88E-04	0.0346	0	EXOC4	GRM7	PDZK1	SNTG2	CCDC88C
GO0002483	6	23	6.59E-04	0.0346	0	TAP2	TAPBP	ERAP1	ERAP2	TRPC4AP
GO0030545	10	28	8.00E-04	0.0394	0	NRG3	NRG1	PPARGC1B	AGRN	ANGPT4
GO0050770	21	56	8.72E-04	0.0406	0	LRRC4C	POU3F2	NRP1	CDH4	NTN1
GO0051338	199	478	9.87E-04	0.0437	0	CD4	C5	PARD3	NRG1	**RAC1**
GO0015399	100	380	0.0011	0.0454	0	TAP2	ABCC4	ATP6V1G2	ATP8B4	ABCC6
GO0006909	26	72	0.0011	0.0454	0	GULP1	ELMO1	MYO7A	ABCA1	CD93
GO0005548	27	63	0.0011	0.0454	0	ATP8B4	CETP	ABCA1	ATP11A	ABCA4
GO0005099	81	256	0.0013	0.0471	0	RASA2	DOCK2	TBC1D10B	TBC1D13	SYDE2
GO0005158	20	51	0.0013	0.0471	0	ENPP1	DOK2	PIK3R1	IRS2	SORBS1
GO0046942	58	149	0.0014	0.0471	0	OCA2	ARL6IP5	SLC3A2	SLC16A1	SLC7A2

* 1347 pathways from BioCarta, KEGG, and GO
** only 5 genes showed for each pathway

Supplement Table 2 GSA-SNP pathway association tests for ADIPOGen Consortium data (corrected *P*<0.05,FDR=0)

pathways*	gene count	set size	*P*	corrected *P*	FDR	gene symbols**					
GO0042058	7	16	0	0	0	**CDH13**	EGF	SNX6	TGFA	EREG	EPGN
vobesityPathway	8	9	0	0	0	ADIPOQ	PPARG	TNF	NR3C1	LPL	RETN
GO0048661	8	19	0	0	0	**CDH13**	CALCRL	TNF	IGF1	HBEGF	NOX1
GO0045296	9	24	0	0	0	**CDH13**	CTNNA3	CTNNA2	CTNNB1	CTNNAL1	P2RX4
GO0001938	10	23	0	0	0	**CDH13**	VEGFA	PDGFB	TNFSF12	ARNT	HIF1A
GO0030100	12	29	0	0	0	**CDH13**	SNX17	RAB5B	TSC2	RAB4A	SYNJ2BP
GO0050920	12	27	0	0	0	**CDH13**	VEGFA	SLIT2	CXCL12	PRKCA	KDR
GO0050921	13	29	0	0	0	**CDH13**	VEGFA	SLIT2	PDGFB	CXCL12	PRKCA
GO0050918	13	29	0	0	0	**CDH13**	VEGFA	TSC2	CXCL12	PRKCA	CORO1A
GO0050850	13	34	0	0	0	**CDH13**	ITGAL	SYK	P2RX2	CD4	ZAP70
GO0030169	16	34	0	0	0	**CDH13**	STAB1	LRP1B	VLDLR	STAB2	COLEC12
GO0016339	19	54	0	0	0	**CDH13**	NLGN1	PCDHGB4	PCDHB2	(PCDHB3)	CDH23
GO0043542	28	67	0	0	0	**CDH13**	VEGFA	CALCA	NF1	MYH9	PDGFB
GO0030335	30	67	0	0	0	**CDH13**	VEGFA	CSF1	RRAS2	PIK3R1	INSR
hsa04930	44	73	0	0	0	ADIPOQ	SLC2A4	CACNA1B	PIK3R1	PRKCE	PKLR
hsa04920	71	105	0	0	0	ADIPOQ	SLC2A4	LEPR	RELA	TNFRSF1B	RXRB
hsa03320	67	93	0	0	0	ADIPOQ	PPARD	PPARG	RXRB	SCP2	HMGCS2
GO0007156	85	343	0	0	0	CDH13	PCDHA1	(PCDHA10)	(PCDHA11)	(PCDHA12)	(PCDHA13)
**GO0032990**	174	414	1.78E-15	1.06E-13	0	CDH13	IGSF9	IFT172	MTSS1	**RAC1**	PCDH15
GO0003689	5	12	1.43E-07	8.04E-06	0	RFC4	RFC5	RFC3	RFC1	RFC2	
iresPathway	7	8	1.36E-05	0.0018	0	EIF4A2	EIF4A1	EIF4G3	EIF3A	EIF4G1	EIF4E

* 1347 pathways from BioCarta, KEGG, and GO

** only 6 genes showed for each pathway
